# Metabolic/endocrine disorders in survivors of childhood-onset and cranial radiotherapy- treated ALL/NHL: a meta-analysis

**DOI:** 10.1186/s12958-023-01137-y

**Published:** 2023-10-04

**Authors:** Dan Zhang, Min Gu

**Affiliations:** 1 Department of Pediatric Endocrine and Metabolism, Shengjing Hospital of China Medical University, Shenyang, China; 2 Department of Pediatrics, Shengjing Hospital of China Medical University, No. 36, Sanhao Street, Heping District, Shenyang, 110004 China

**Keywords:** Acute lymphoblastic leukemia, Non-hodgkin’s lymphoma, metabolic/endocrine disorders, Cranial radiotherapy, Meta-analysis

## Abstract

**Background:**

Cranial radiotherapy (CRT) is recommended to high-risk pediatric patients with acute lymphoblastic leukemia or aggressive non-Hodgkin’s lymphoma (ALL/NHL). However, effects of CRT treatment on the development of metabolic/endocrine disorders remain unclear. This meta-analysis aimed to identify metabolic and endocrine disturbances in survivors of childhood-onset and CRT-treated ALL/NHL.

**Methods:**

Different online databases were searched using restricted search fields. Follow-up data and outcome measurements, including the prevalence of growth hormone (GH) deficiency, hypothyroidism, vitamin D deficiency, overweight/obesity, and hypogonadism were recorded. The height data was indicated by height-standard deviation score (height-SDS). Statistical estimates such as odds ratio (OR) and weighted standard mean difference (SMD) were compared between additional CRT treatment group and non-CRT treatment group. Study-to-study heterogeneity was calculated by calculating I-squared statistic, and fixed/random effect was applied to synthesize and analyze extracted data.

**Results:**

Fifteen studies were included (4269 patients in total). Adult height SDS was lower in CRT-treated patients (pooled SMD = -0.581, 95% CI: -0.649–-0.512), and CRT-treated patients were likely to develop short stature (pooled OR = 2.289, 95% CI:1.674–3.130). Regardless of the study year, which potentially reflects the state-of-the-art CRT technique, the prevalence of short stature and GH deficiency was time-independent. Additionally, previous CRT can increase the risk of precocious puberty (pooled OR = 2.937, 95% CI: 1.281–6.736), hypothyroidism (pooled OR = 2.057, 95% CI:1.510–2.801), and hypogonadism (pooled OR = 3.098, 95% CI:2.521–3.807). However, the risk of being overweight/obese was similar between the patients with and without CRT (pooled OR = 1.278, 95% CI: 0.675–2.421).

**Conclusion:**

Childhood-onset and CRT-treated ALL/NHL survivors are likely to have shorter height, precocious puberty, hypothyroidism, and hypogonadism.

## Background

Endocrine complications are common in the treatment of acute lymphoblastic leukemia or non-Hodgkin’s lymphoma (ALL/NHL) and may weaken patients during and after treatment. Metabolic disturbances or endocrine disorders in survivors of childhood-onset ALL/NHL are mostly attributed to cytotoxic medication [1] which can directly and widely injure cell types in the course of development and differentiation. Glucocorticoids play an indispensable role in the endocrine system by disturbing endogenous hormone secretion, glycolipid-protein metabolism, and electrolyte balance [1, 2]. Additionally, radiotherapy, including total body and regional radiotherapy, can cause complicated endocrine problems [3] and increase the 3-year incidence of gonadal and growth hormone deficiencies [4]. Testicular radiation can influence semen quality and fertility [5]. Moreover, cranial radiotherapy (CRT) is essential for patients with secondary cranial metastatic lesions or those at high risk of cranial involvement [6]. However, CRT treatment can inevitably cause side effects to intracranial structures, such as the pituitary and hypothalamus, which lie at the core position of the endocrine axis [7, 8]. A slow growth rate is common during the treatment period, including chemotherapy and radiotherapy, in pediatric patients with ALL/NHL [1, 9]. However, children who do not receive head irradiation usually reach a normal height in adulthood [10]. Although growth hormone deficiency usually occurs in patients receiving CRT at a radiation dose of 24 Gy, it may also occur in patients receiving low-dose (18 Gy) CRT or high-dose methotrexate [1]. However, the effect of CRT on height in ALL/NHL survivors remains unclear. Additionally, CRT can influence thyroid [11] and gonadal function [5, 12]. Additionally, metabolic problems such as obesity, being overweight, vitamin D deficiency, and osteopenia have an ambiguous relationship with CRT treatment [13–16]. With prolonged survival, the toxicity and side effects of CRT treatment may increase. However, the effects of CRT treatment on the development of metabolic and endocrine disorders remain unclear. Therefore, in this meta-analysis, we aimed to investigate the influence of CRT on metabolic/endocrine disorders in survivors of childhood-onset ALL/NHL in terms of body stature, body weight, thyroid function, and gonadal function.

## Methods

### Retrieval from online databases

Information from online databases (PubMed, Web of Science, Google Scholar, and the Cochrane Library) was retrieved, and keywords, including pediatric patient, child patient, acute lymphoblastic leukemia, non-Hodgkin’s lymphoma, metabolic/endocrine disorders, cranial radiation therapy, radiotherapy, and irradiation were used to search for literature published from the database’s inception to September, 1, 2022. The full search string across different electronic databases based on the Boolean logic operator was “(pediatric patient + child patient) * (acute lymphoblastic leukemia + non-Hodgkin’s lymphoma) * (metabolic disorders + endocrine disorders) * (cranial radiation therapy + radiotherapy + irradiation).”

### Study inclusion, quality assessment, and data extraction

Two researchers searched the online database independently based on the following inclusion/exclusion criteria: (1) an observational study design aiming to compare the prognostic difference in patients with or without previous CRT treatment in advance; (2) previously treated survivors of pediatric ALL/NHL; (3) patients with previous CRT treatment as the intervention group; and (4) patients without previous CRT treatment as the control group. Outcome measurements at the last visit were classified into discontinuous and continuous variables. Discontinuous variables included the prevalence of short stature, growth hormone (GH) deficiency, precocious puberty, hypothyroidism, vitamin D deficiency, overweight/obesity, and hypogonadotropic hypogonadism (hypogonadism). Height data were indicated by the height-standard deviation score (height-SDS) as a continuous variable. Statistical estimates such as the odds ratio (OR) and weighted standard mean difference (SMD) were compared between the additional CRT treatment group and the non-CRT treatment group. The quality of the included clinical studies was evaluated using the Newcastle-Ottawa Scale (NOS) on a seven point-scale (Table 1). Two independent investigators reached a consensus on the eligibility of included studies for further meta-analyses. The outcomes were defined as follows: short stature was defined as height ≤ − 2SD. Precocious puberty was defined as the onset of puberty (breast budding in females, testicular volume > 4 mL in males) before 8 years in girls and 9 years in boys, and elevated plasma gonadotropin hormone concentrations after gonadotropin-releasing hormone (GnRH) stimulation with elevated plasma levels of sex hormones. GH deficiency was defined as peak GH < 10 µg/L until 2011 and thereafter (following a change in the laboratory measurement method) as peak GH < 7.5 µg/L in response to stimulation with clonidine hydrochloride (0.15 mg/m^2^) or glucagon (30 µg/kg). The diagnosis of GH deficiency was based on growth velocity/growth data and low levels of GH in two stimulation tests or low levels of GH in one stimulation test in those who were previously treated with CRT. In this study, hypogonadism refers to hypogonadotropic hypogonadism and was defined as the lack of an increase in serum luteinizing hormone (LH) levels after exogenous GnRH stimulation and sex hormone levels in the prepubertal range (estradiol < 20 pmol/L in females, testosterone < 0.7 nmol/L in males), or decreased levels of estradiol in adult females and testosterone in adult males interpreted according to their age. The GnRH stimulation test (50 µg/m^2^) was performed in patients with failed spontaneous puberty, pubertal arrest, or precocious puberty. Obesity was defined as BMI ≥ 1.645 SD (≥ 95th percentile for age and sex) and overweight as BMI = 1.036–1.644 SD (85th–94.9th percentile for age and sex).

**Table 1:** Quality assessment of prospective cohort studies

Author	Prospective design	Clear definition of study population	(1)	(2)	(3)	(4)	(5)
Elitzur, et al. 2017	No	Yes	Yes	Yes	Yes	Yes	Yes
Bayram et al. 2017	No	Yes	Yes	Yes	Yes	Yes	Yes
Steffens et al. 2008	No	Yes	Yes	Yes	Yes	Yes	Yes
Alves et al. 2004	No	Yes	Yes	Yes	Yes	Yes	Yes
Ghassemi et al. 2016	No	Yes	Yes	Yes	Yes	Yes	Yes
Vilela et al. 2013	No	Yes	Yes	Yes	Yes	Yes	Yes
Chow et al. 2013	No	Yes	Yes	Yes	Yes	Yes	Yes
Hata et al. 2001	No	Yes	Yes	Yes	No	Yes	Yes
Krawczuk-Rybak et al. 2019	No	Yes	Yes	Yes	Yes	Yes	Yes
Piette et al. 2020	No	Yes	Yes	Yes	Yes	Yes	Yes
Cicognani et al. 1992	No	Yes	Yes	Yes	No	Yes	Yes
Shimazaki et al. 2020	No	Yes	Yes	Yes	Yes	Yes	Yes
Adan et al. 2001	No	Yes	Yes	Yes	No	Yes	Yes
Costin et al. 1988	No	Yes	Yes	Yes	No	Yes	Yes
Siimes et al. 1993	No	Yes	Yes	Yes	Yes	Yes	Yes

(1) if the study design was proper or not; (2) was measurement of each outcome indicator stable or not; (3) was CRT plus group and non-CRT group comparable between each other; (4) was there any clear definition of outcome event; (5) was follow-up duration clearly given

### Statistical analysis

The extracted clinical data were analyzed using Stata statistical software (version 13.0; Stata Corporation, College Station, TX, USA). If a study did not include the outcome of interest, it was excluded from the meta-analysis. The heterogeneity among included studies was evaluated using the Q chi-square test, and a p-value < 0.10 was considered to indicate significant heterogeneity in data. The percentage of variability that was attributed to heterogeneity across the studies was assessed using the I^2^ statistic, which indicated variation in different estimates (OR and SMD values) attributable to heterogeneity. Studies with an I^2^ of less than 50% were considered to have no or a low degree of heterogeneity, and vice versa. Pooled estimate statistics (pooled OR and SMD values) were calculated using a fixed-effects method for studies with no or a low degree of heterogeneity. Otherwise, the random-effects method was applied when significant heterogeneity was detected. A sensitivity analysis was performed to explore the potential sources of heterogeneity. The pooled risk of some events was expressed as an odds ratio (OR) with a 95% confidence interval (95% CI). Publication bias was evaluated using Egger’s test. Statistical significance was defined as a two-tailed p-value < 0.05. The study year could potentially reflect the state-of-the-art CRT technique, and improved CRT techniques over time might influence the prevalence of short stature, growth hormone (GH) deficiency, precocious puberty, hypothyroidism, vitamin D deficiency, overweight/obesity, and hypogonadism. Therefore, the time-dependent effects of CRT on different outcomes were confirmed using meta-regression.

## Results

### Literature retrieval, characteristics of included studies and patients’ baseline demography

**Fig. 1:**
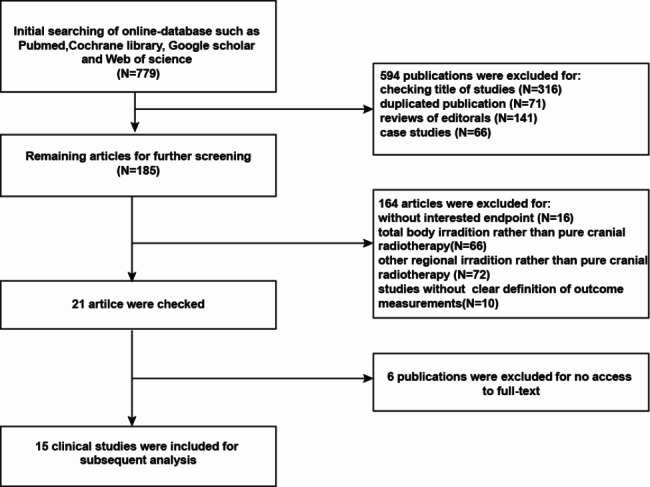
Flow chart of literature retrieval and study inclusion

In the initial retrieval of the academic databases, 779 studies were scanned for their titles, and 594 studies were excluded as duplicates, study types that did not comply with the meta-analysis or obvious irrelevancy with the research objective of the present meta-analysis. The abstract of each retrieved article was carefully checked, and 170 studies were filtered out for different reasons, such as mechanistic studies based on basic research, studies without outcomes of interest, no access to full-text manuscripts, interventional studies instead of observational studies, or case studies. Finally, 15 studies [17–31] were included; a flowchart of study selection is shown in Fig. 1. Four single-arm studies were included for a meta-regression analysis to analyze the time-dependent effect of CRT treatment on different outcomes. The characteristics of the included studies and the patients’ demography at baseline are shown in Table 1, including author information, study year, study region, number of patients, study design, number of cohorts, hematologic neoplasm type, follow-up duration (years), male proportion (%), age at diagnosis of ALL/NHL (years), proportion of prepubertal stage at diagnosis (%), median age at the last visit, number of patients receiving CRT (%), and CRT doses. Clinical data from 4269 patients in the included studies were integrated and analyzed. In general, the follow-up duration in the included studies was approximately one decade. The ultimate quality check of the included clinical studies using NOS is shown in Table 1. Two independent investigators reached a consensus that the included studies were eligible for further meta-analyses.

### CRT in childhood influencing survivors’ height in the follow-up duration

**Fig. 2:**
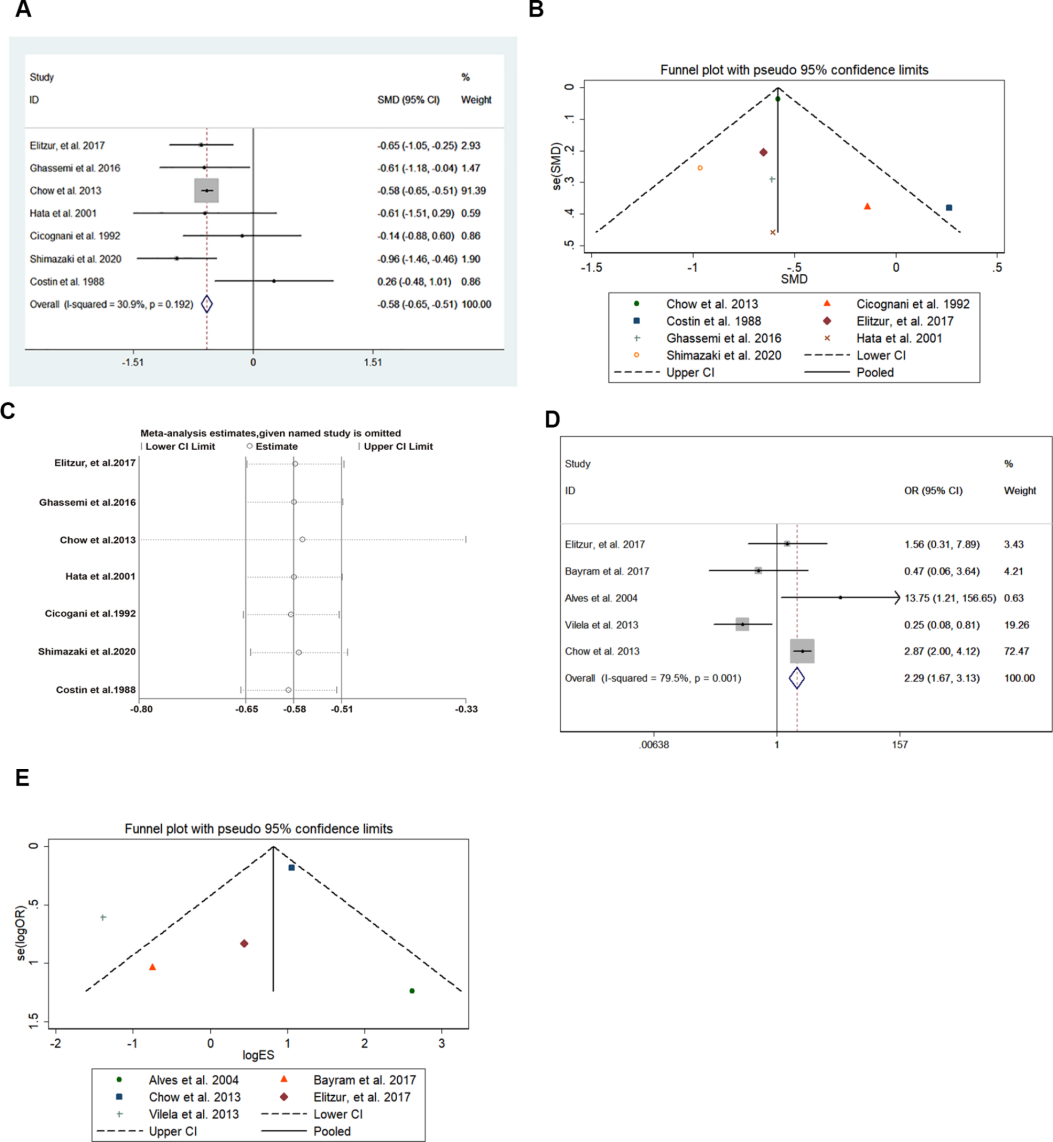
CRT in childhood influencing survivors’ height during follow-up. Value of height-SDS (**A**); funnel plot showing the symmetric distribution of included studies in this analysis (**B**); sensitivity analysis indicating no inter-publication heterogeneity (**C**); CRT treatment as risk factors in developing a short stature (**D**); publication bias analysis by funnel plot (**E**)

**Fig. 3:**
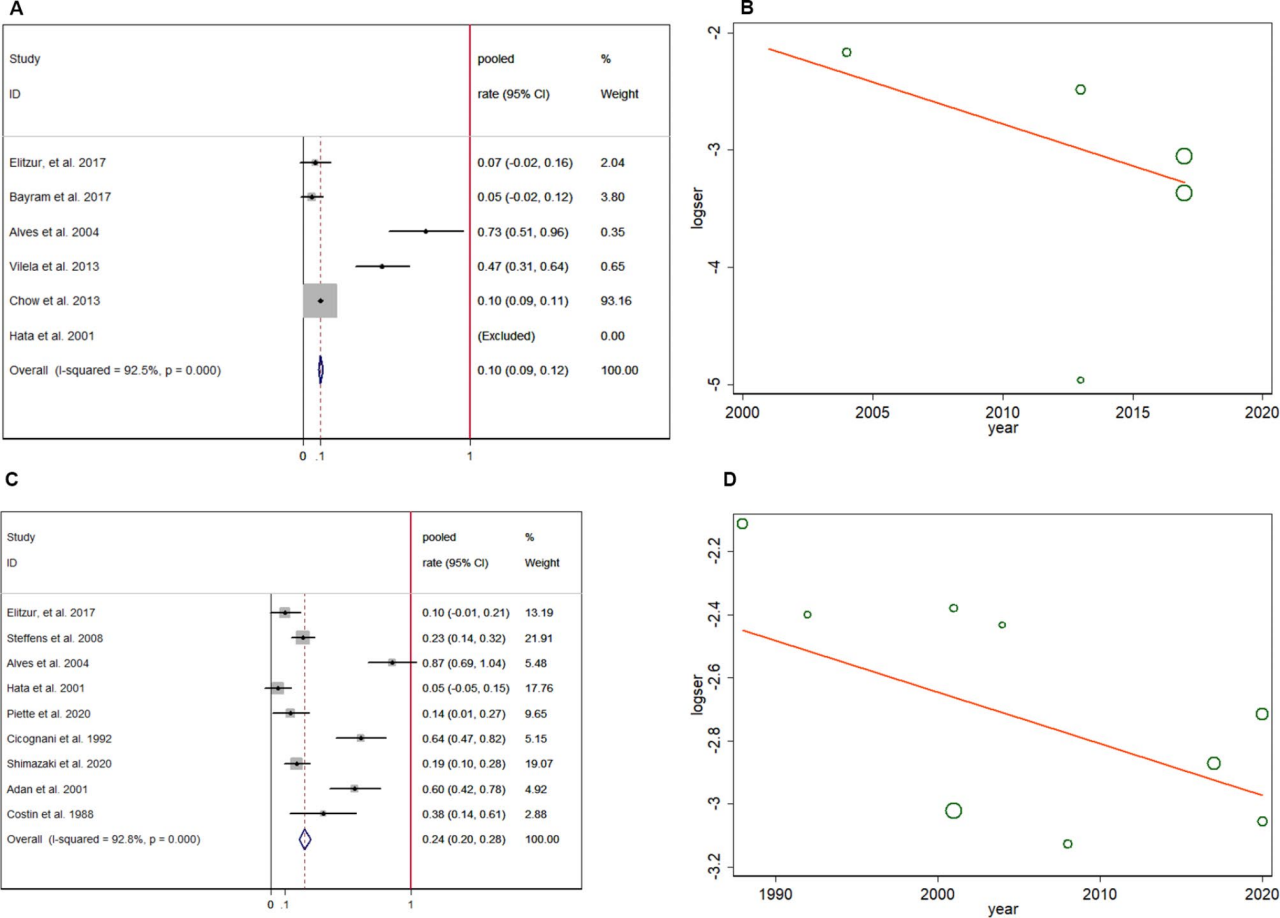
Pooled prevalence of short stature in included patients (**A**); meta-regression of time influence on short stature prevalence (**B**); pooled GH deficiency rate (**C**); meta-regression of time influence on the prevalence of GH deficiency (**D**)

Seven related studies were included in the analysis of adult height SDS (3724 patients) [17, 18, 22, 24, 27, 28, 30]. Figure 2 A shows that the adult height SDS was lower in CRT-treated patients (pooled SMD = -0.581, 95% CI: -0.649–-0.512). No heterogeneity was found in the extracted data (I-squared = 30.9%, p = 0.192), and the symmetric distribution of the included studies in the funnel plot (Fig. 2B) revealed no publication bias (p > 0.05). Moreover, sensitivity analysis, shown in Fig. 2C, suggests no inter-publication heterogeneity, and thus, no further subgroup analysis was required. Patients receiving CRT in childhood were likely to develop short stature (pooled OR = 2.289, 95% CI:1.674–3.130) in the follow-up period (Fig. 2D), and significant heterogeneity among the included studies was detected (I-squared = 79.5%, p = 0.001). The funnel plot in Fig. 2E shows no publication bias (P > 0.05). Six related studies were included in the analysis of the short stature (3716 patiens) [17–19, 21, 23, 24]. In patients with a history of CRT, the pooled prevalence rate of short stature was 0.103 (95% CI:0.09–0.116), as shown in Fig. 3A. Meta-regression was performed to analyze the correlation between short stature and different study years to test whether the proportion of patients with short stature in CRT-treated patients changed over time. The meta-regression coefficient between short stature and different study years was 0.931 (95% CI:0.703–1.233), indicating no time effect in CRT-treated patients who finally developed short stature (Fig. 3B). In addition, the GH secretion level, which was positively correlated with height, was assessed in 631 patients, and the pooled GH deficiency rate was 0.243 (95% CI:0.203–0.284) (Fig. 3C) [17, 18, 20, 21, 26–30]. The study year, which potentially reflects the state-of-the-art CRT technique, showed no relationship with GH deficiency prevalence by meta-regression (Fig. 3D), with a coefficient of 0.984 (95% CI:0.927–1.044). The importance of distinguishing GHD short stature from non-GHD short stature should also be considered. However, due to the lack of data on GH treatment in patients with GHD, this comparison could not be performed.

### Precocious puberty in ALL/NHL survivors who received CRT treatment in childhood

**Fig. 4:**
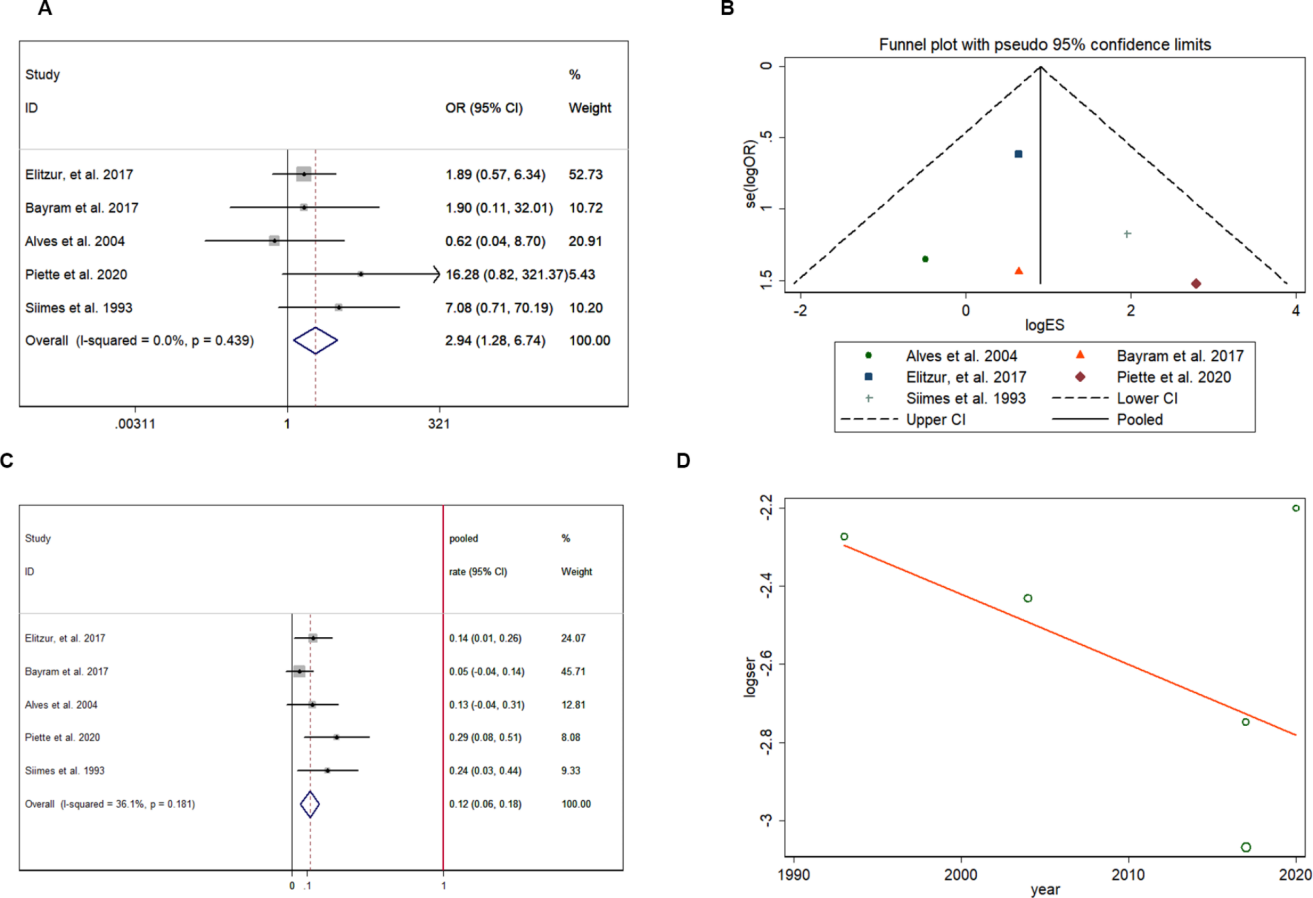
Precocious puberty in ALL/NHL survivors who received CRT treatment in childhood: CRT increases the risk of precocious puberty (**A**); funnel plot showing no publication bias (**B**); pooled prevalence of precocious puberty (**C**); meta-regression of time influence on precocious puberty (**D**)

**Fig. 5:**
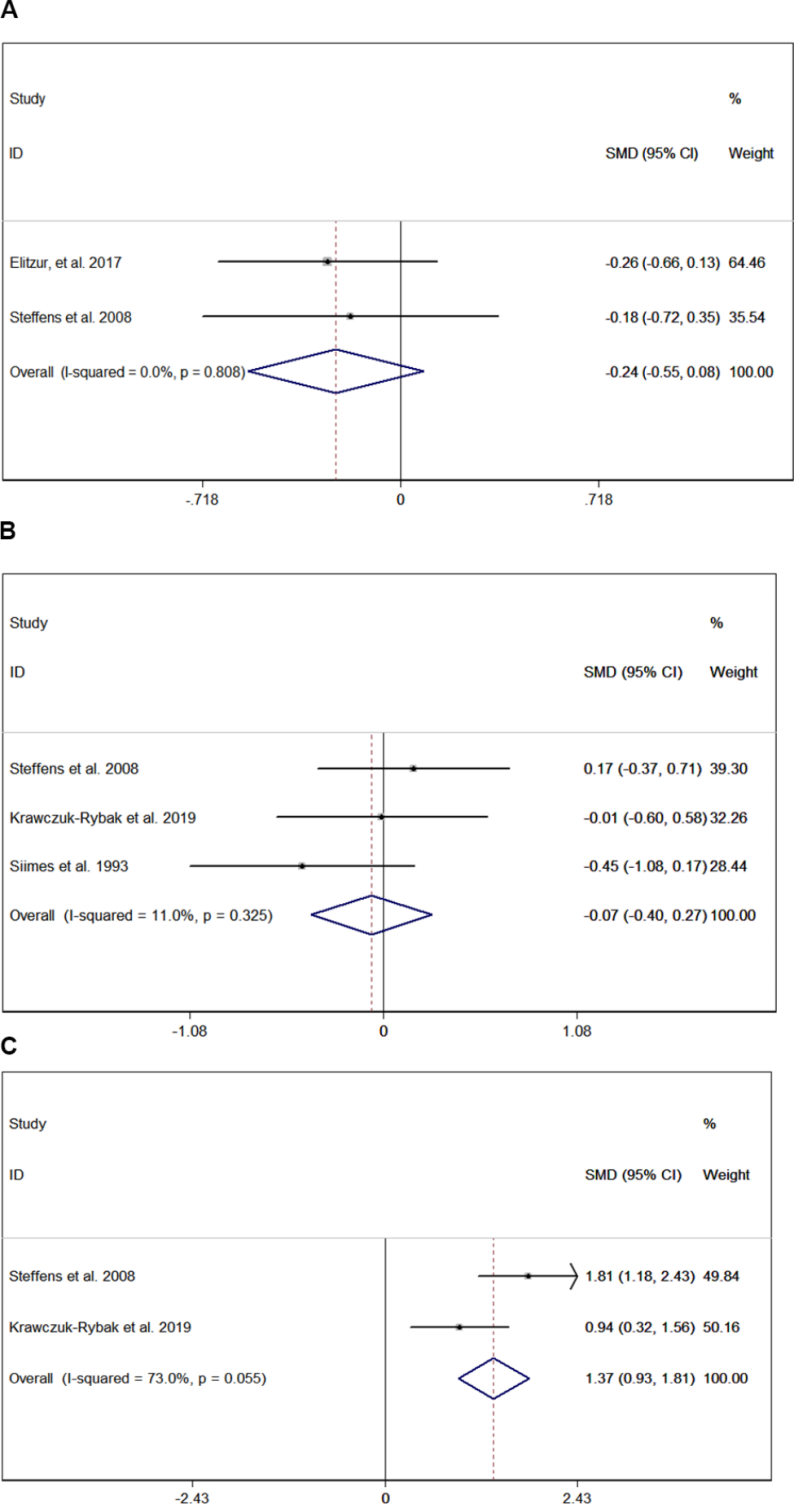
Difference in mean age at menarche (**A**); difference in testosterone level (**B**); difference in estradiol level (**C**)

Five studies with 478 patiens were included in the analysis of precocious puberty [18, 19, 21, 26, 31]. CRT could increase the risk of precocious puberty (pooled OR = 2.937, 95% CI:1.281–6.736) in ALL/NHL survivors who received CRT treatment previously (Fig. 4A), and no heterogeneity was found in extracted data (I-squared < 1%, p = 0.439). In the funnel plot shown in Fig. 4B, the included studies were symmetrically distributed, indicating no publication bias (p > 0.05). In patients with previous CRT treatment, the pooled prevalence rate of precocious puberty was 11.8% (95% CI, 0.056–0.179; Fig. 4C). Meta-regression analysis (Fig. 4D) showed that the study year was not correlated with the prevalence of precocious puberty, with a coefficient of 0.982 (95% CI:0.889–1.084). Especially in female patients, the age at menarche tended to be earlier (pooled SMD = -0.235, 95% CI: -0.555–0.084) in patients with a history of CRT treatment (Fig. 5A), and no heterogeneity was found in extracted data (I-squared < 1%, p = 0.808). Additionally, the effects of CRT on sex hormone levels were evaluated. The testosterone level (nmol/L) showed no difference (pooled SMD = -0.065, 95% CI: -0.401 to 0.271) regardless of CRT treatment (Fig. 5B), and no heterogeneity was found in the extracted data (I-squared = 11.0%, p = 0.325). However, estradiol (ng/mL) was higher in patients previously treated with CRT (pooled SMD = 1.372, 95% CI:0.93–1.814), as shown in Fig. 5C.

### Hypothyroidism and hypogonadism after CRT treatment

**Fig. 6:**
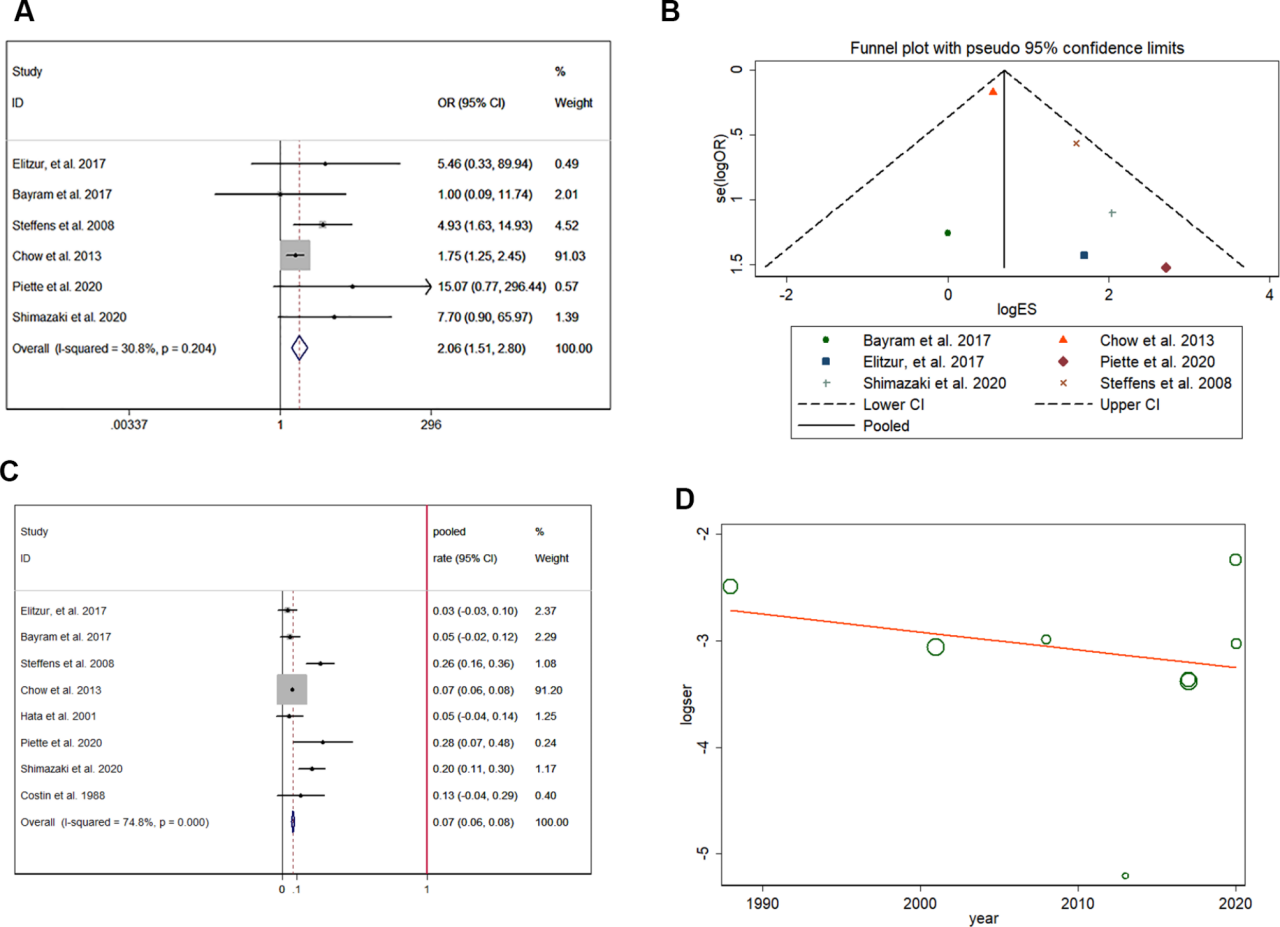
Hypothyroidism and hypogonadism after CRT treatment: increased risk of hypothyroidism after CRT treatment (**A**); funnel plot showing a symmetric distribution of included studies in this analysis (**B**); pooled prevalence rate of hypothyroidism (**C**); meta-regression of time influence on hypothyroidism (**D**)

**Fig. 7:**
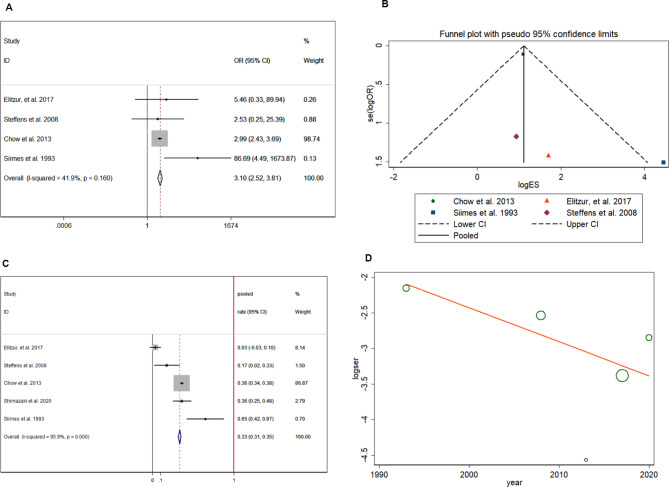
Increased risk of hypogonadism after CRT treatment (**A**); symmetrical distribution of included studies indicating no publication bias (**B**); pooled prevalence rate of hypogonadism (**C**); meta-regression of time influence on hypogonadism (**D**)

In analysis of 3907 patients, the risk of hypothyroidism was significantly increased (pooled OR = 2.057, 95% CI:1.510–2.801) in ALL/NHL survivors who had previously received CRT (Fig. 6A) [18–20, 24, 26, 28], and no heterogeneity was found in the extracted data (I-squared = 30.8%, p = 0.204). In the funnel plot shown in Fig. 6B, the included studies were symmetrically distributed, indicating no publication bias (p > 0.05). In patients with previous CRT treatment, the pooled prevalence rate of hypothyroidism was 7.1%, with a 95% CI ranging from 6.1 to 8.1% (Fig. 6C) [17–20, 24, 28, 30] after analyzing data from 3784 patients. Meta-regression (Fig. 6D) showed that the study year did not correlate with the prevalence of hypothyroidism with a coefficient of 0.983 (95% CI:0.931–1.039). However, the risk of hypogonadism was also significantly increased (pooled OR = 3.098, 95% CI:2.521–3.807) in ALL/NHL survivors who had previously received CRT (Fig. 7A) [18, 20, 24, 31], and no heterogeneity was found in the extracted data (I-squared = 41.9%, p = 0.16) from 3645 patients. In the funnel plot shown in Fig. 4F, the included studies are symmetrically distributed, indicating no publication bias (p > 0.05). In patients with previous CRT, the pooled prevalence rate of hypogonadism was 33.3%, with a 95% CI ranging from 31.4 to 35.2% (Fig. 7B). Meta-regression (Fig. 7C) [18, 20, 24, 31] showed that the study year did not correlate with the prevalence of hypogonadism with a coefficient of 0.953 (95% CI:0.827–1.098) in included 3645 patients. Distinguishing between TSH deficiency (CRT) and primary hypothyroidism (neck irradiation) is important. However, the influence of radiation therapy on hypothyroidism could not be compared because of the lack of relevant data.

### Other metabolic/endocrine problems in survivors after CRT in childhood

**Fig. 8:**
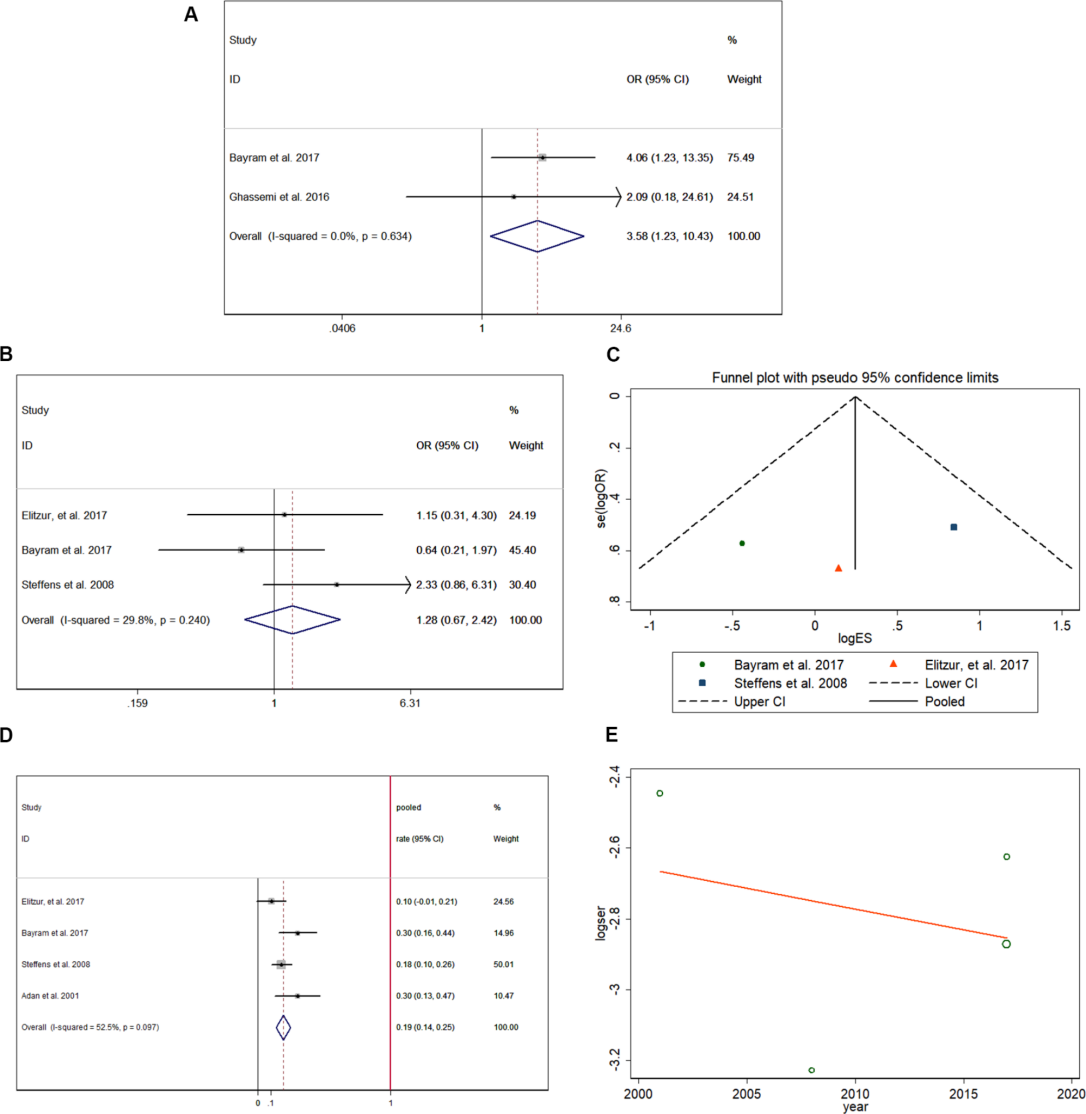
Other metabolic/endocrine problems of survivors after CRT in childhood: risk of vitamin D deficiency after CRT treatment (**A**); risk of being overweight/obese after CRT treatment (**B**); funnel plot showing symmetric distribution of included studies in this analysis (**C**); pooled prevalence rate of being overweight/obese (**D**); meta-regression of time influence on being overweight/obese (**E**)

According to the abovementioned CRT-related short stature, the risk of vitamin D deficiency was higher (pooled OR = 3.575, 95% CI:1.226–10.427) in patients who had previously received CRT (Fig. 8A) [19, 22] from 110 patients, and no heterogeneity was found in the extracted data (I-squared < 0.1%, p = 0.634). The risk of overweight/obesity showed no difference (pooled OR = 1.278, 95% CI:0.675–2.421) between CRT-treated and non-CRT-treated patients (Fig. 8B) [18–20], and no heterogeneity was found in the extracted data (I-squared = 29.8%, p = 0.24) from 271 patients. In the funnel plot shown in Fig. 8C, the included studies are symmetrically distributed, indicating no publication bias (p > 0.05). By analyzing data reported from 349 patients with previous CRT treatment, the pooled prevalence rate of being overweight/obese was 19.2%, with a 95% CI ranging from 13.7 to 24.7% (Fig. 8D) [18–20, 29]. Meta-regression (Fig. 8E) showed that the study year did not correlate with being overweight/obese with a coefficient of 0.988 (95% CI:0.758–1.289).

## Discussion

Here, we aimed to reveal metabolic and endocrine disturbances in survivors of childhood-onset and CRT-treated ALL/NHL. Typically, the follow-up duration of the included studies was approximately one decade. In this study, adult height-SDS was lower in CRT-treated patients (pooled SMD = -0.581, 95% CI: -0.649–-0.512), and CRT-treated patients were likely to develop short stature (pooled OR = 2.289, 95% CI:1.674–3.130). Regardless of the study year, which potentially reflects the state-of-the-art CRT technique, the prevalence of short stature and GH deficiency was time-independent. Additionally, previous CRT increased the risk of precocious puberty (pooled OR = 2.937, 95% CI,1.281–6.736), hypothyroidism (pooled OR = 2.057, 95% CI,1.510–2.801), and hypogonadism (pooled OR = 3.098, 95% CI,2.521–3.807). However, the risk of being overweight/obese was similar (pooled OR = 1.278, 95% CI:0.675–2.421) between patients with and without CRT. Based on the abovementioned evidence, CRT treatment could lead to metabolic and endocrine disorders in survivors of childhood-onset ALL/NHL.

To date, most pediatric patients with ALL/NHL undergo stratified treatment according to risk factors. However, the toxicity and side effects of related treatments, such as CRT, have been shown to increase with prolonged survival. Most ALL/NHL-related endocrine complications are caused primarily by ALL therapy. Endocrine complications may affect pediatric patients, acting as an acute response in the early phase or a persistent response in the delayed phase after comprehensive antitumor treatment [1]. However, Elitzur et al. reported that most childhood ALL survivors receiving chemotherapy alone attained normal adult height and puberty [18]. Therefore, the role of radiotherapy in treatment-related endocrine disorders requires further investigation. CRT may be the major risk factor for final height deficit [23]. Ghassemi et al. evaluated the influence of chemotherapy plus radiotherapy on bone mineral density (BMD) in pediatric patients with ALL/NHL and argued that 94% of the included patients had abnormal bone density [22]. These studies suggest that CRT is correlated with bone metabolism, which potentially leads to short stature and osteoporosis. GH replacement therapy is necessary to prevent and correct height and bone density deficits in certain cases [32]. The safety and efficacy of GH replacement therapy have been assessed and validated for the correction of GH deficiency in survivors of childhood ALL [33]. GH replacement therapy in survivors of childhood-onset ALL treated with prophylactic CRT for GH deficiency improves cardiac systolic function and reduces the prevalence of metabolic syndrome [34]. Therefore, monitoring the GH level and growth velocity in pediatric ALL/NHL patients after CRT and evaluating the indications for GH replacement therapy are important. It is also important to distinguish between short stature with and without GH deficiency. However, no information on factors that could influence short stature was provided in the included studies. Therefore, we speculate that CRT might be related to, rather than lead to, short stature. This potential bias has been discussed further.

Young survivors of childhood-onset ALL, especially those treated with cranial irradiation, are at high risk for developing obesity, dyslipidemia, insulin resistance, hypertension, and full dysmetabolic syndrome early after the completion of therapy [35]. Although there is sufficient evidence that obesity worsens ALL outcomes [36], clinical and preclinical data indicate that ALL/NHL and its related treatment might influence body weight [37]. In a prospective obesity intervention trial (NCT02708108), metabolic intervention, which included a 10% dietary calorie restriction and a 10% increase in energy consumption through exercise, significantly decreased the incidence of body weight and obesity. Most importantly, this intervention reduced fat accumulation and the incidence of minor residual disease, indicating that it could improve the efficacy of chemotherapy in pediatric cases of ALL [38]. However, our study suggests that CRT does not influence the prevalence of being overweight or obese. We hypothesized that overweight/obesity is multifactorial determined, and personal lifestyle and eating habits could significantly influence its outcome. Further understanding of the biological mechanisms of obesity could provide a new perspective for the development of treatment-related obesity and comprehensive methods for its prevention. Bayram et al. observed a high frequency of endocrine complications in a clinical study and identified vitamin D insufficiency/deficiency (46.6%), overweight/obesity (33.3%), short stature (6.7%), thyroid function abnormality (5%), and precocious puberty (3.3%) within a median follow-up time of four years [19]. Their discovery is in accordance with the findings of this meta-analysis. Moreover, our study validates that the prevalence of endocrine complications is time-independent. In addition to endocrine disorders, long-term CRT negatively affects cognitive function in children [39].

Finally, the limitations of this meta-analysis need to be acknowledged. Two studies included patients with both NHL and ALL. However, a subgroup analysis was not performed in a limited number of studies, and the difference in the effects of CRT on patient prognosis between NHL and ALL could not be determined from this meta-analysis. According to the characteristics of the included studies, there was no randomization design, which reduced the evidence quality of our meta-analysis. As shown in Table 1, the CRT radiation dose in the included studies ranged from 10 to 58 Gy, which could lead to bias and heterogeneity. Subgroup analyses based on different CRT radiation doses could not be performed because of limited access to more specific data in most studies. Whether CRT has a dose-dependent effect on the prognosis of pediatric patients warrants further investigation in future studies. In general, the follow-up duration in the included studies was approximately one decade. However, the assessment of changes over chronological time (years) could not be analyzed and adjusted for the limitation of access to the data of specific follow-up durations in the included studies. In this study, only hypogonadotropic hypogonadisms instead of hypergonadotropic hypogonadisms, however, it was partly owing to anti-tumor therapy used in included studies was not “high-risk” (such as melphalan, busulphan, thiotepa, and no stem cell transplant, etc.). In addition, we cannot differentiate hypogonadotropic hypogonadisms from hypergonadotropic hypogonadisms, since there is no access to acquiring data to evaluate levels of LH and follicle stimulating hormone. Of the total number of 4,269 included patients, 3,343 were from one study (Chow et al. 2013) and this issue needs to be addressed in more detail. Some outcomes (excluding this study) were based on a small number of participants. This is an important limitation of this study.

## Conclusion

Patients treated with CRT in childhood tended to have lower adult-height SDS and were likely to develop short stature. Regardless of the study year, which potentially reflects the state-of-the-art CRT technique, the prevalence of short stature and GH deficiency was time-independent. Additionally, previous CRT was found to increase the risk of precocious puberty, hypothyroidism, and hypogonadism. However, the risk of being overweight/obese was similar between the patients with and without CRT. Survivors of childhood-onset and CRT-treated pediatric patients with ALL/NHL were likely to have shorter height, precocious puberty, hypothyroidism, and hypogonadism. CRT and CRT-related endogenous hormone disturbances may be responsible for these metabolic and endocrine disorders.

## Data Availability

The datasets used and/or analyzed in this study are available from the corresponding author upon reasonable request.
